# Supplementary material for: Spatial coordination in a mutually beneficial bacterial community enhances its antibiotic resistance
Source: Commun Biol. 2019 Aug 8;2:301. doi: 10.1038/s42003-019-0533-0 (PMC6687750; doi:10.1038/s42003-019-0533-0)
Supplement: Supplementary file 1 — Supplementary Information [file 42003_2019_533_MOESM1_ESM.pdf]

Supplementary Table 1 Strains and plasmids used in this study.

	Genotype	Phenotype or Description	Source or Reference
Plasmids			
pUA66-CTX-M-14	KAN^r CRO^r GFP	Expresses GFP proteins and β-lactam antibiotic hydrolase (CTX-M)	[3]
BBa_J61002	AMP^r mCherry	Expresses mCherry proteins and an ampicillin-resistance gene（TEM）	From iGEM parts
pUA66	KAN^r GFP	Expresses GFP proteins and a kanamycin-resistance gene	[3]
E. coli strain			
DH5α	F- φ80 lacZΔM15 Δ(lacZYA-argF) U169 recA1 endA1 hsdR17 (rk-, mk+) phoA supE44 thi-1 gyrA96 relA1 λ-	Cannot use lactose	[3]
MG1655	F- λ- ilvG- rfb-50 rph-1	Can use lactose	From the Peking University iGEM team (Haoqian Zhang)
BW25113	Δ(araD-araB)567, ΔlacZ4787(::rrnB-3), lambda-, rph-1, Δ(rhaD-rhaB)568, hsdR514	Cannot use lactose but has other characteristics similar to those of MG1655.	From the Peking University iGEM team (Haoqian Zhang)
Resistant bacteria		DH5α with pUA66-CTX-M-14; shows resistance to ceftriaxone sodium (abbreviated as CRO).	
Nutritional bacteria		MG1655 with BBa_J61002; shows resistance to ampicillin (abbreviated as Amp) and uses lactose as food.	
Sensitive bacteria		DH5α with pUA66; shows resistance to kanamycin (abbreviated as Kan).	
Non-nutritional bacteria		BW25113 with BBa_J61002; shows resistance to Amp.	

Supplementary Table 2 Growth of the different strains in different media (in standard tubes).

Strain	Medium	
	LB + CRO	M9 + lactose
Resistant bacteria	Growth	Death or no growth
Nutritional bacteria	Death or no growth	Growth
Sensitive bacteria	Death or no growth	Death or no growth
Non-nutritional bacteria	Death or no growth	Death or no growth

Supplementary Table 3 Appropriate culture medium for different strains (in standard tubes).

Strain	Medium
Resistant bacteria	LB + 2 μg mL^{-1} CRO
Nutritional bacteria	LB + 250 μg mL^{-1} Amp
Sensitive bacteria	LB + 50 μg mL^{-1} Kan
Non-nutritional bacteria	LB + 250 μg mL^{-1} Amp

Supplementary Table 4 Growth results of mixed cultures in the chips (OD_{600}=10, in chips). Only mutualistic communities can grow in the medium supplemented with CRO and with lactose as the only carbon source.

Strains	Growth Result	Image (0 h)	Image (20 h)
Resistant bacteria + nutritional bacteria	Growth	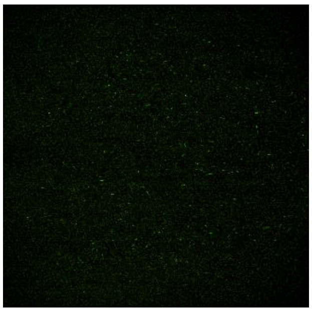	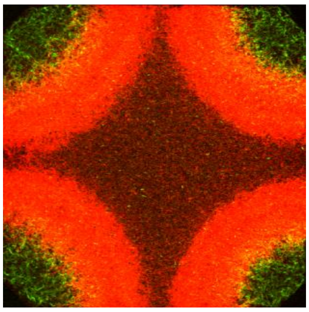
Resistant bacteria + non-nutritional bacteria	Death or no growth	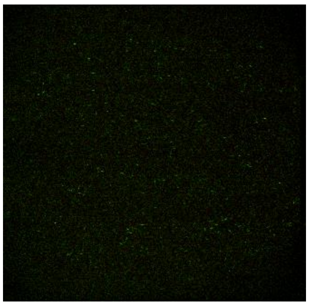	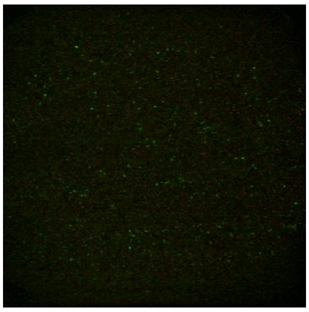
Sensitive bacteria + nutritional bacteria	Death or no growth	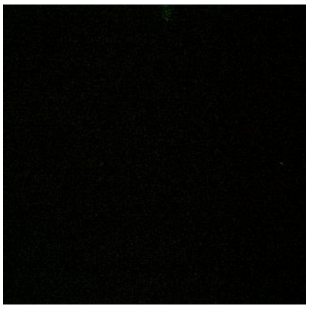	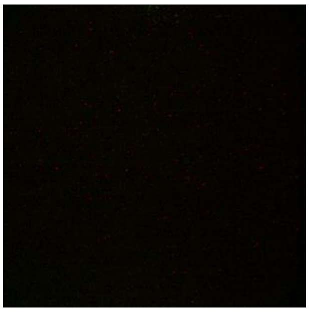
Sensitive bacteria + non-nutritional bacteria	Death or no growth	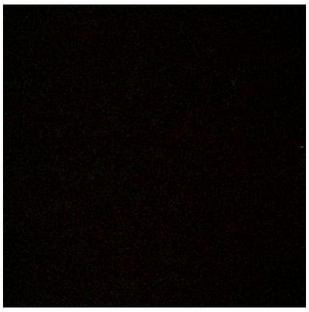	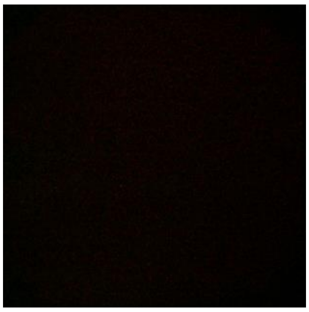

Note: The images are superimposed with the GFP and mCherry channels, and the LUTs of the eight images are consistent. The resistant and sensitive bacteria are labeled with GFP, and the nutritional and non-nutritional bacteria are labeled with RFP. The medium used was M9 containing 0.8% lactose, 2000 μg mL^{-1} CRO and 250 μg mL^{-1} Amp.

Supplementary Table 5 Growth of resistant bacteria in the presence of nutritional bacteria (in chips, without CRO).

Strains	Growth state	Image (0 h)	Image (20 h)
Resistant bacteria	Death or no growth	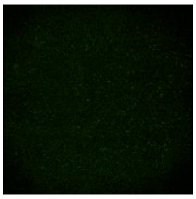	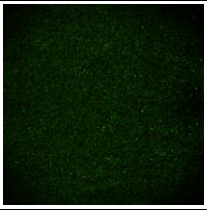
Nutritional bacteria	Growth	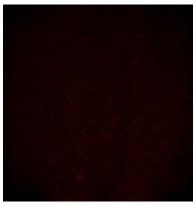	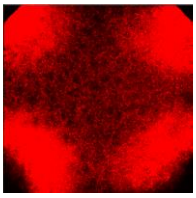
Resistant bacteria + nutritional bacteria	Growth of both strains	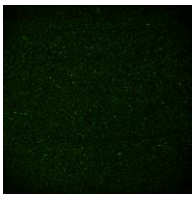	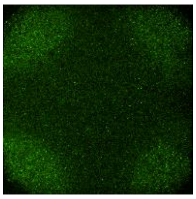
		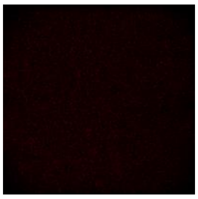	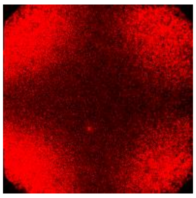
		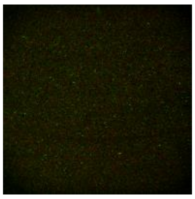	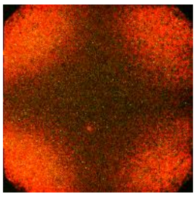

Note: The images are superimposed with the GFP and mCherry channels, and the LUTs of the images taken at the same time are consistent. The resistant bacteria are labeled with GFP, and the nutritional bacteria are labeled with RFP. The medium used was M9 containing 0.8% lactose and 250 μg mL^{-1} Amp. Images of co-cultured sample are represented by each single fluorescent channel and a combination of two channels.

Supplementary Table 6 Parameters in the simulation model

Parameter	Definition	Value
k_1	Maximal growth rate of Y1 (s^{-1})	0.0001
k_2	Maximal mortality rate of Y1 (s^{-1})	0.0003
k_4	Maximal growth rate of Y2 (s^{-1})	0.0002
k_5	Maximal mortality rate of Y2 (s^{-1})	0.0003
K_1	Characteristic nutrient concentration for Y1	2
K_2	Characteristic antibiotic concentration for Y1	50
K_4	Characteristic nutrient concentration for Y2	0.1
K_5	Characteristic antibiotic concentration for Y2	500
c_1	Maximal consumption rate of N1 (s^{-1})	0.0005
C_1	Characteristic concentration of N1	2
c_2	Maximal consumption rate of N2 or N3 (s^{-1})	0.001
C_2	Characteristic concentration of N2 or N3	0.1
c_3	Maximal consumption rate of A (s^{-1})	0.02
C_3	Characteristic concentration of A	5
D_{N1}	Diffusion coefficient of N1 ($\mu m^2\ s^{-1}$)	430
D_{N2}	Diffusion coefficient of N2 ($\mu m^2\ s^{-1}$)	1800
D_{N3}	Diffusion coefficient of N3 ($\mu m^2\ s^{-1}$)	80
D_A	Diffusion coefficient of A ($\mu m^2\ s^{-1}$)	350
λ_{N2}	Conversion rate of N2	0.1
λ_{N3}	Conversion rate of N3	0.8
R	Nutrients contained in a single bacterium	3.47
N_{max}	Maximal number of bacteria in each grid	9600

Most parameters have been verified by other experiments [2,3].

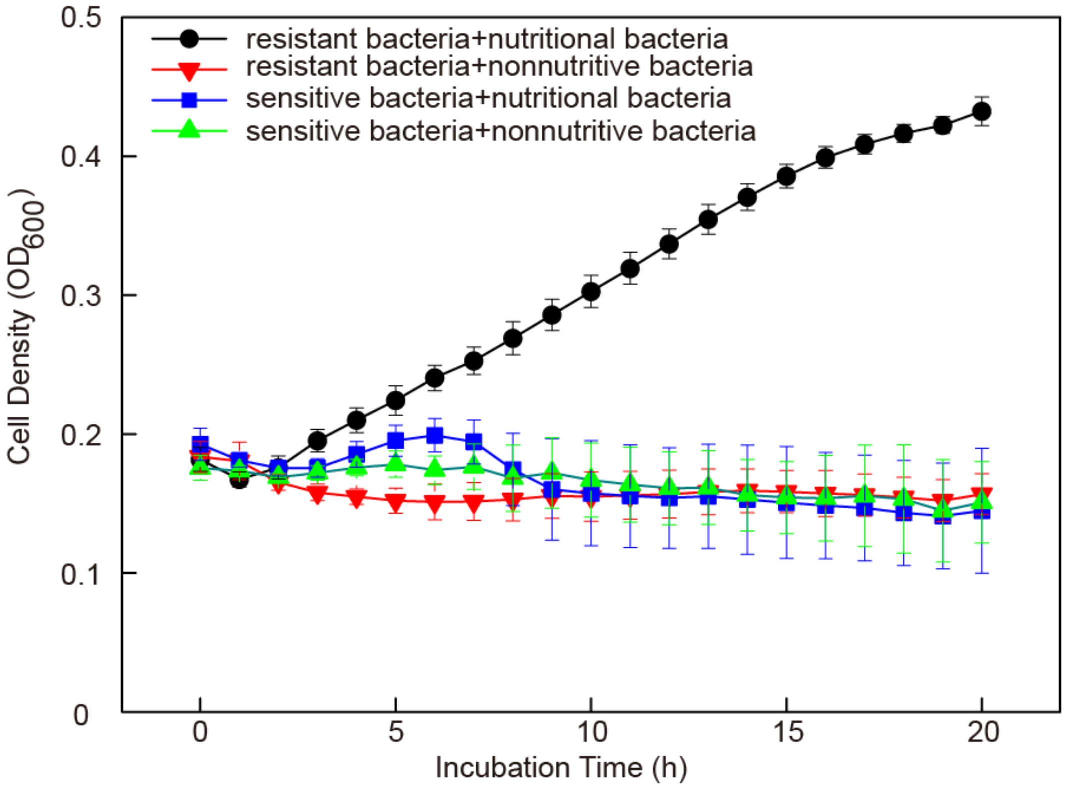

Supplementary Figure 1 Growth of the mixed cultures. The four samples shown in the picture were grown under the same experimental conditions with the exception of the type of bacteria. Equal amounts of the two bacteria were mixed evenly and cultured in a tube with M9 medium containing 100 μg mL^{-1} CRO, 250 μg mL^{-1} Amp and 0.8% lactose. After culture for a certain time, the OD_{600} of the culture was measured using a microplate reader. The dots showed the averages of seven to eight repeated experiments and the whiskers indicated the standard deviation. Only the coculture of resistant and nutritional bacteria showed obvious growth.

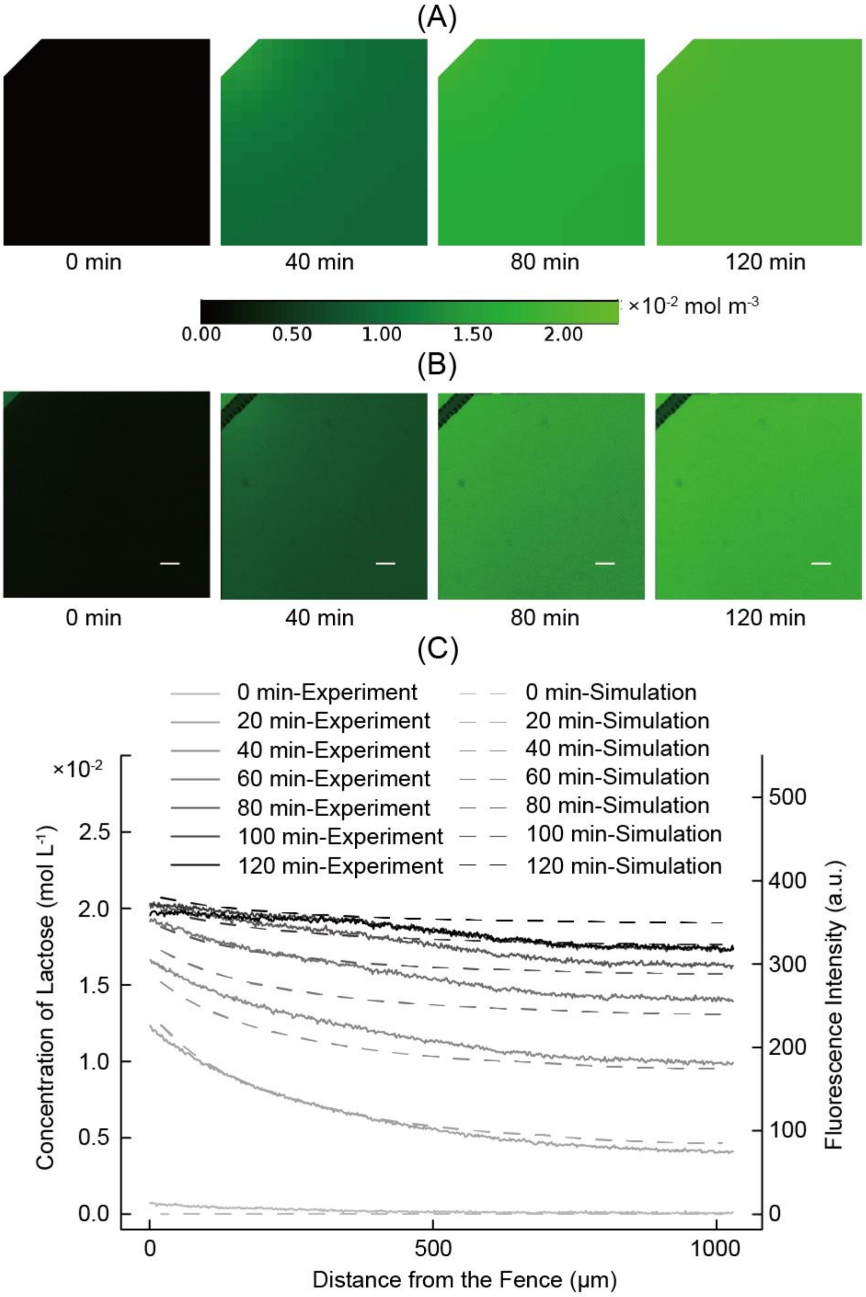

Supplementary Figure 2 MATLAB simulation and experiment for processing the diffusion of lactose/fluorescein in the chip. (*A*) The diffusion of lactose in the chip was simulated by MATLAB, and a quarter of the culture area was used for display purposes. The upper left corner shows the fence and diffusion source. The lactose in the chip became almost uniform within approximately 100-120 min. (*B*) The diffusion of lactose in the chip was verified by fluorescein, which has a similar diffusion coefficient to that of lactose. A quarter of the fluorescence image was also used for the display. The upper left panel shows the reservoir. The diffusion of fluorescein was consistent with the simulation results. Scale bars: 100 μm. (*C*) The fluorescence intensity on the diagonal of the simulation results and the experimental images at each time point were extracted, and an image in which the fluorescence intensity changes over time was then obtained. The dotted lines show the simulation results, and the solid lines represent the experimental results.

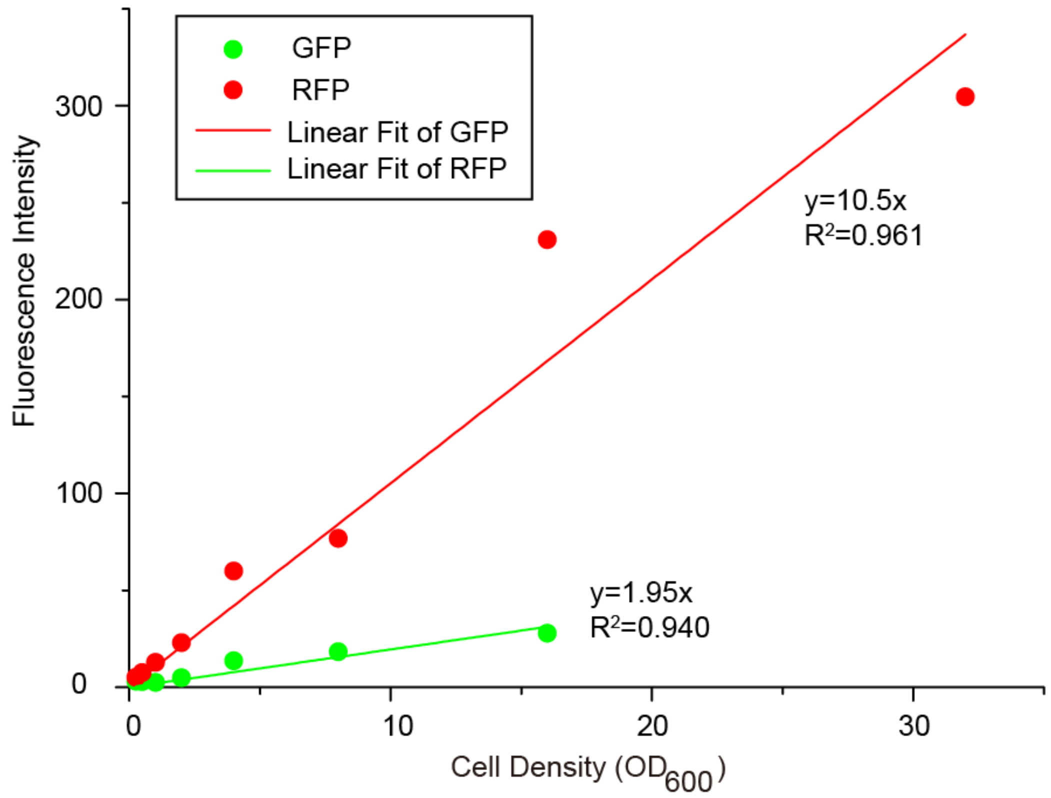

Supplementary Figure 3 Linear relationship between the bacterial density and fluorescence intensity. The abscissa shows the density of the culture added to the chip, and the ordinate is the average fluorescence intensity obtained from the analysis of the micrographs of the region with bacteria. The red dots indicate the nutritional bacteria, and the green dots indicate the resistant bacteria. The linear fitting results for each dataset are drawn with a straight line, and the specific information is shown in the table. The linear relationship between the bacterial density and average fluorescence intensity is good.

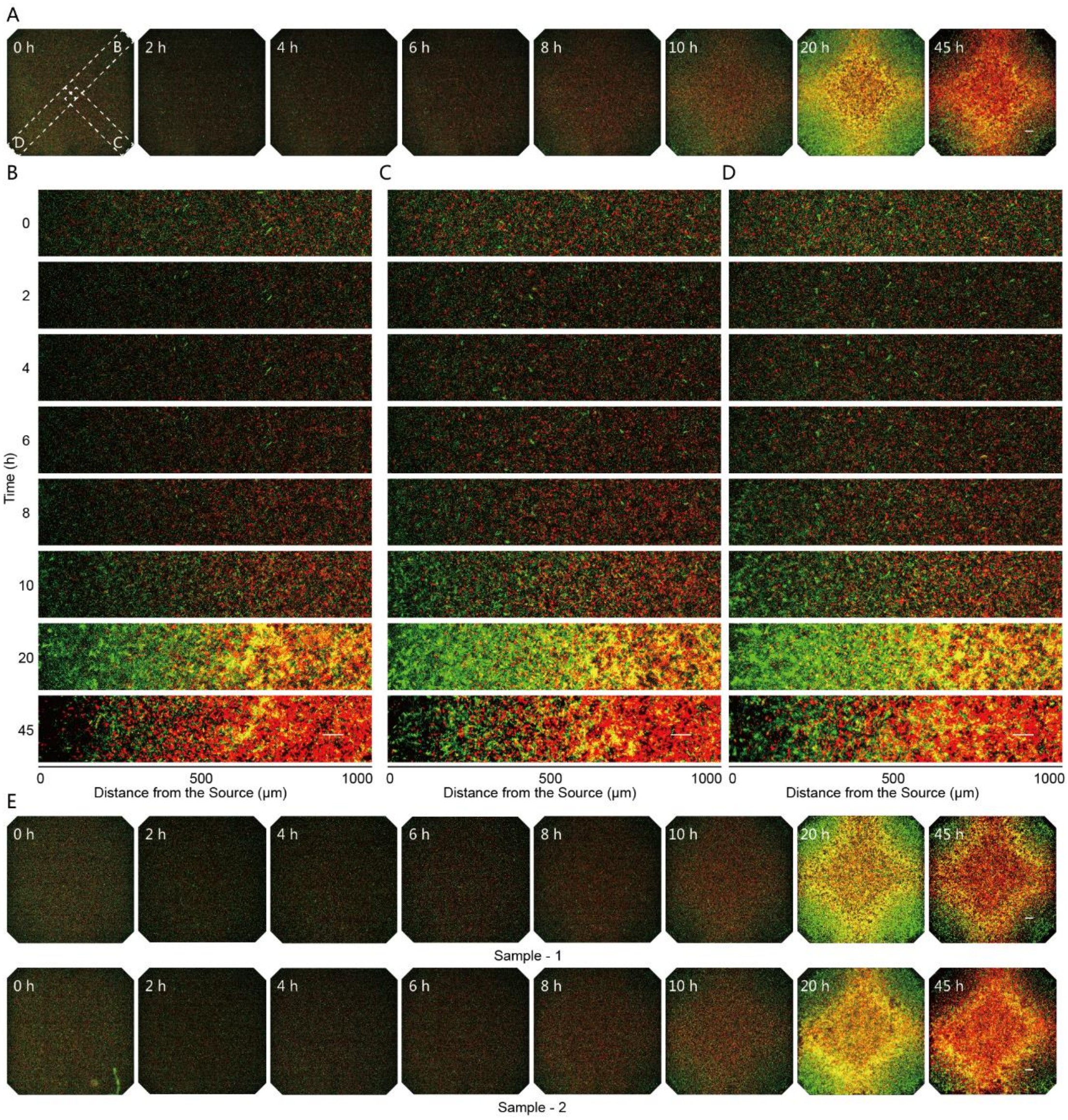

Supplementary Figure 4 (A) Time series of micrographs of one sample (Fig. 3 shows one diagonal line of the sample). (B, C, D) The other three diagonal lines of the same sample as shown in Fig. S4 (A). (E) The time series of micrographs of two additional samples. Scale bars: 100 μm.

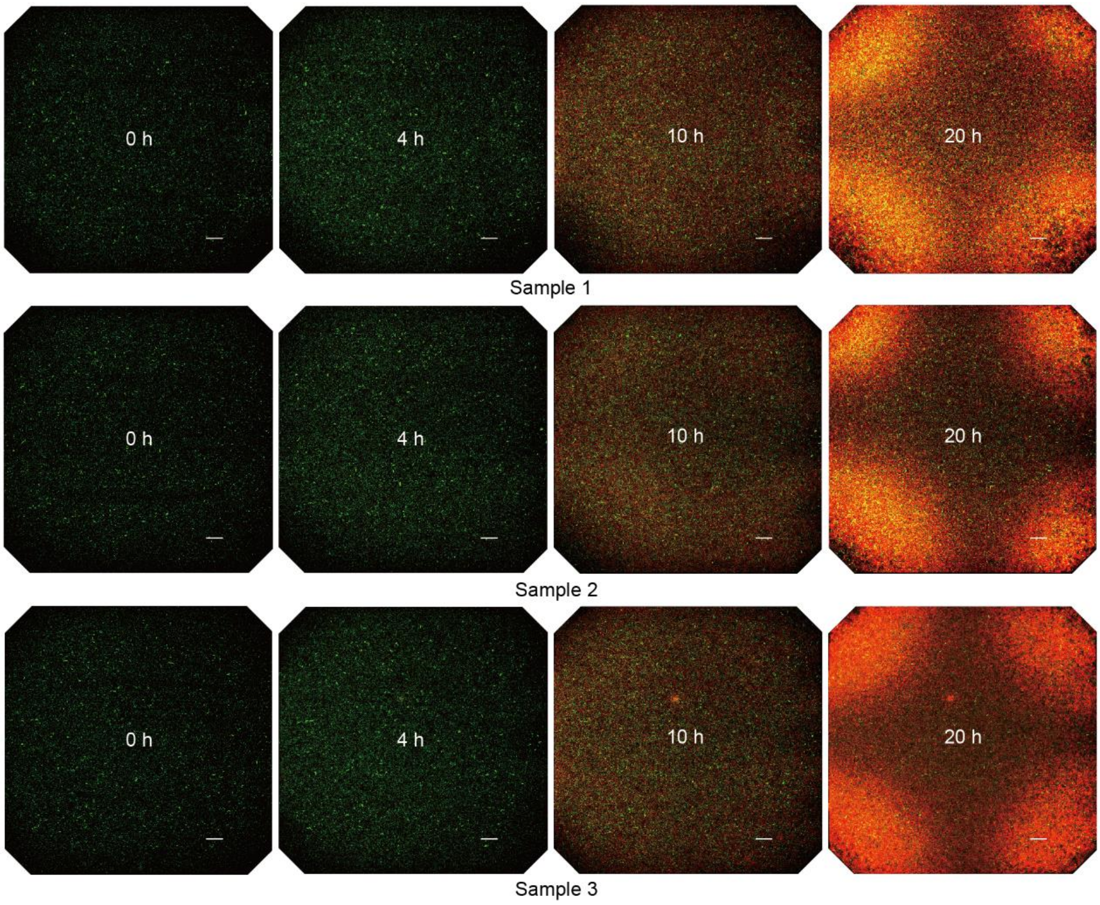

Supplementary Figure 5 Time series of three repeated experiments with 0 μg mL^{-1} CRO (parasitic communities). Scale bars: 100 μm.

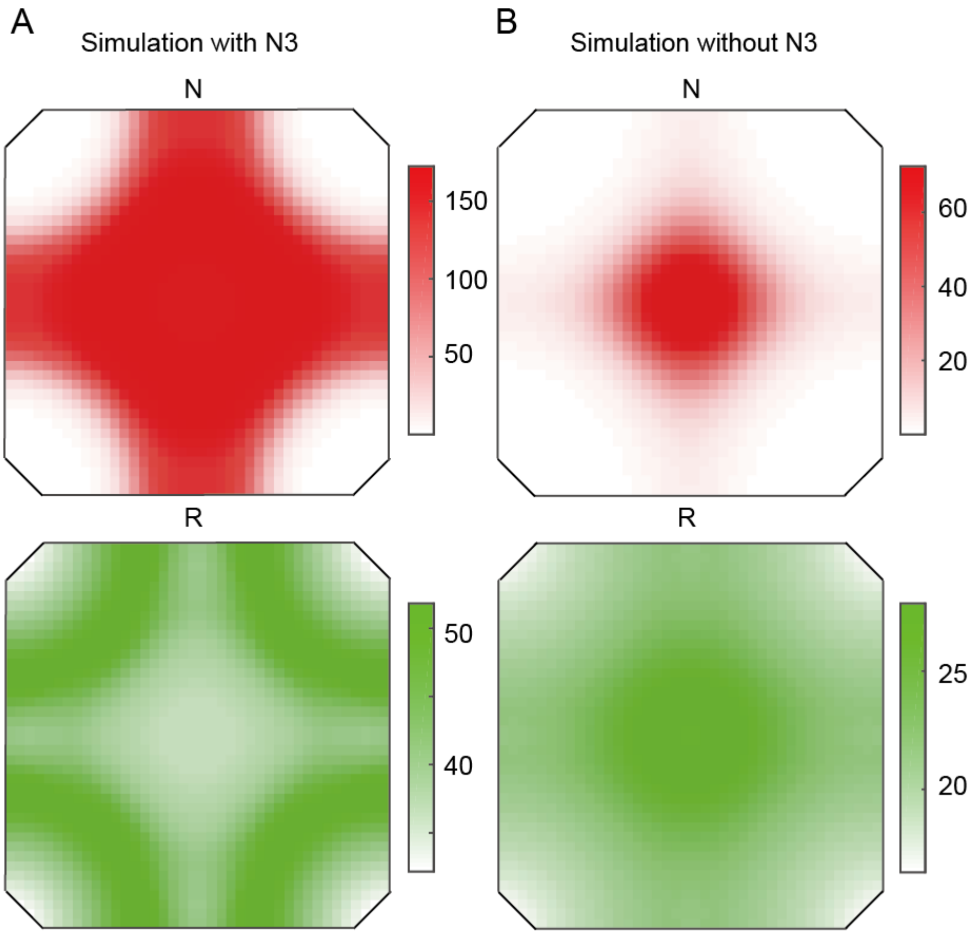

Supplementary Figure 6 Comparison of the simulation results with and without N3. N3 is the nutrient released by dead bacteria that can be used by living bacteria. The figure shows the spatial distributions of the two bacteria in the community with an initial density of OD_{600} 2.5 after 10 h of culture. The red and green colors indicate the nutritional and resistant bacteria, respectively, and the depth of the color indicates the density of the bacteria.

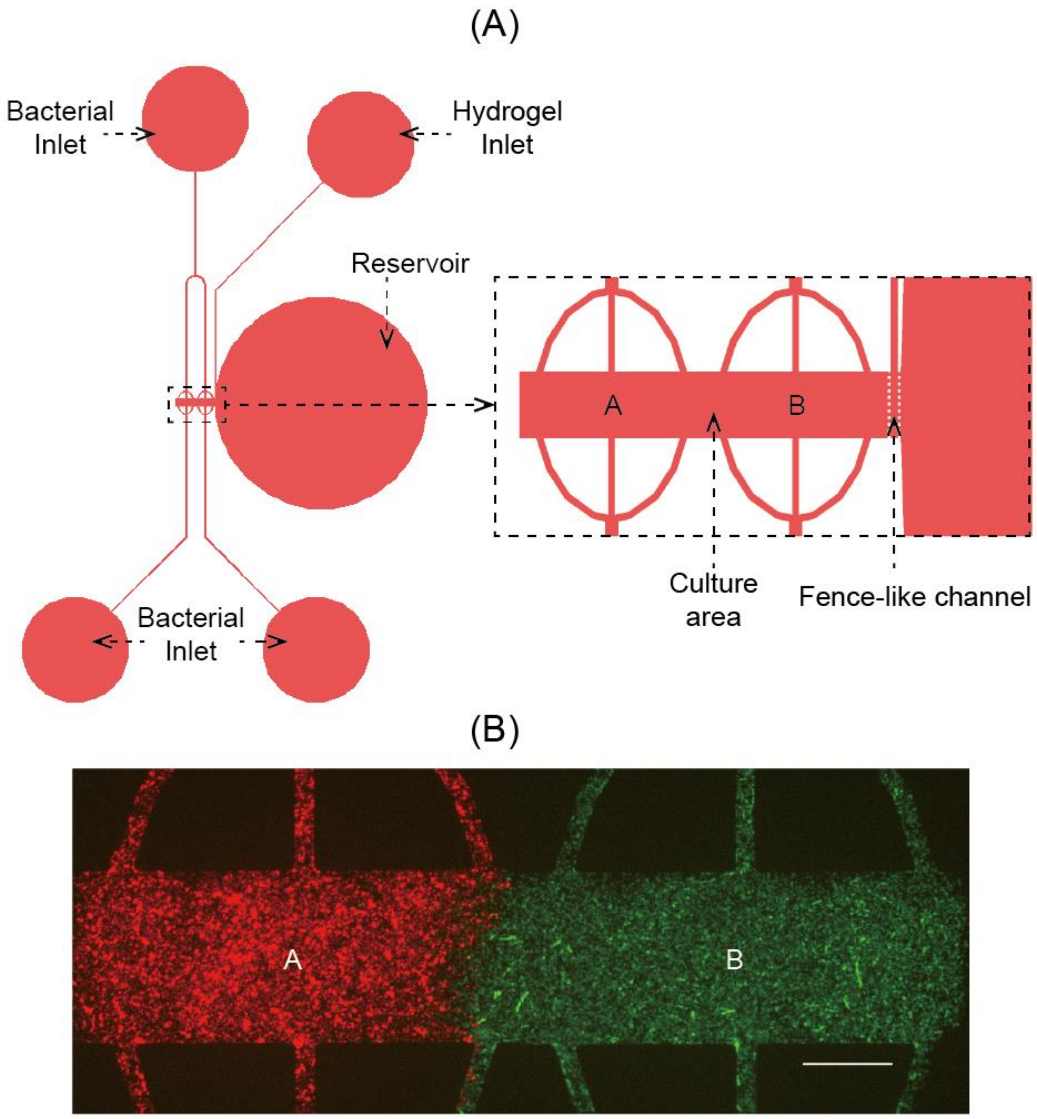

Supplementary Figure 7 Design and application of the stripe chip for controlling the initial distribution. (A) Design of the stripe chip. The left picture shows the entire chip, and the right picture shows an enlarged view of the culture area. The functions of the reservoir, fence and culture area of the stripe chip are the same as those of the square chip. The unique function of the stripe chip is that different initial bacterial distributions can be obtained by adding different bacteria to regions A and B in the culture region. (B) Micrograph of the initial distribution obtained using the stripe chip. Region A contains only nutritional bacteria, and only resistant bacteria are found in region B. Both types of bacteria are evenly distributed and well separated from each other. Scale bars: 100 μm.

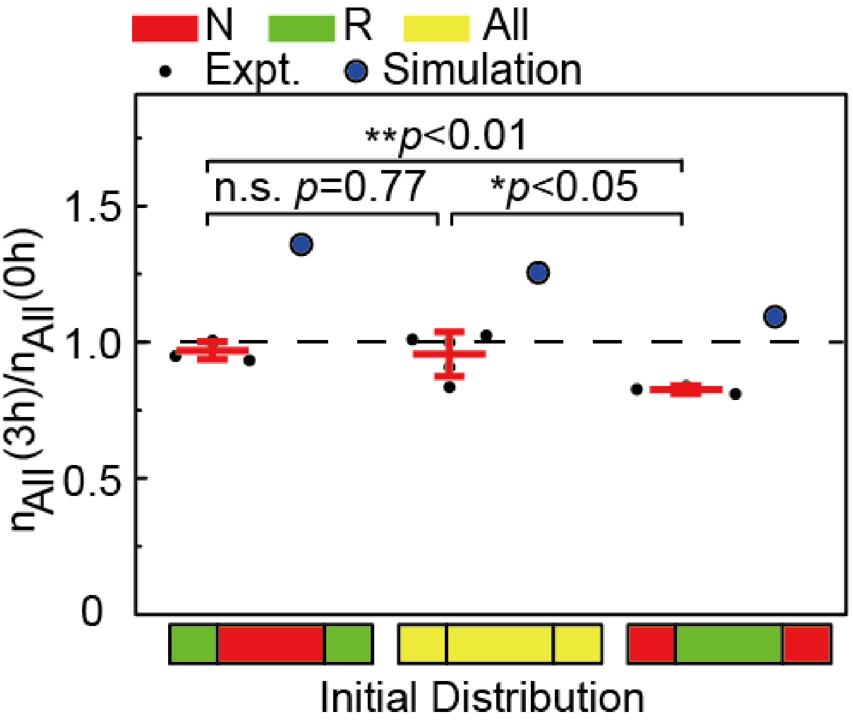

Supplementary Figure 8 Communities with the same initial density show different survival rates depending on the initial distribution. The three rectangles drawn below the histogram represent the three initial distributions. The red or green colors indicate the presence of only nutritional or resistant bacteria, respectively, and the yellow color represents a homogeneous mixing of the two bacteria. The ordinate shows the community survival rate at 3 h. Error bars represent standard error of the mean. The asterisk indicates the significance of the difference between the samples in each group. "*" indicates that the P value of the t test is less than 0.05, and "**" indicates that the P value of the t test is less than 0.01. There are 3 to 5 replicate samples in each group.

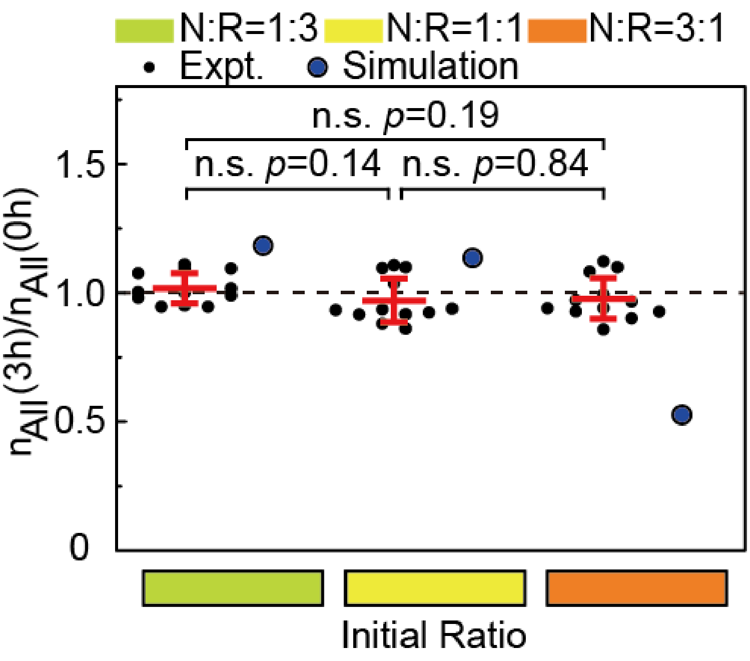

Supplementary Figure 9 Communities with the same initial density show different survival rates depending on the initial N/R ratio. The three rectangles drawn below the histogram represent the three ratios tested. The ordinate shows the community survival rate at 3 h. Error bars represent standard error of the mean.

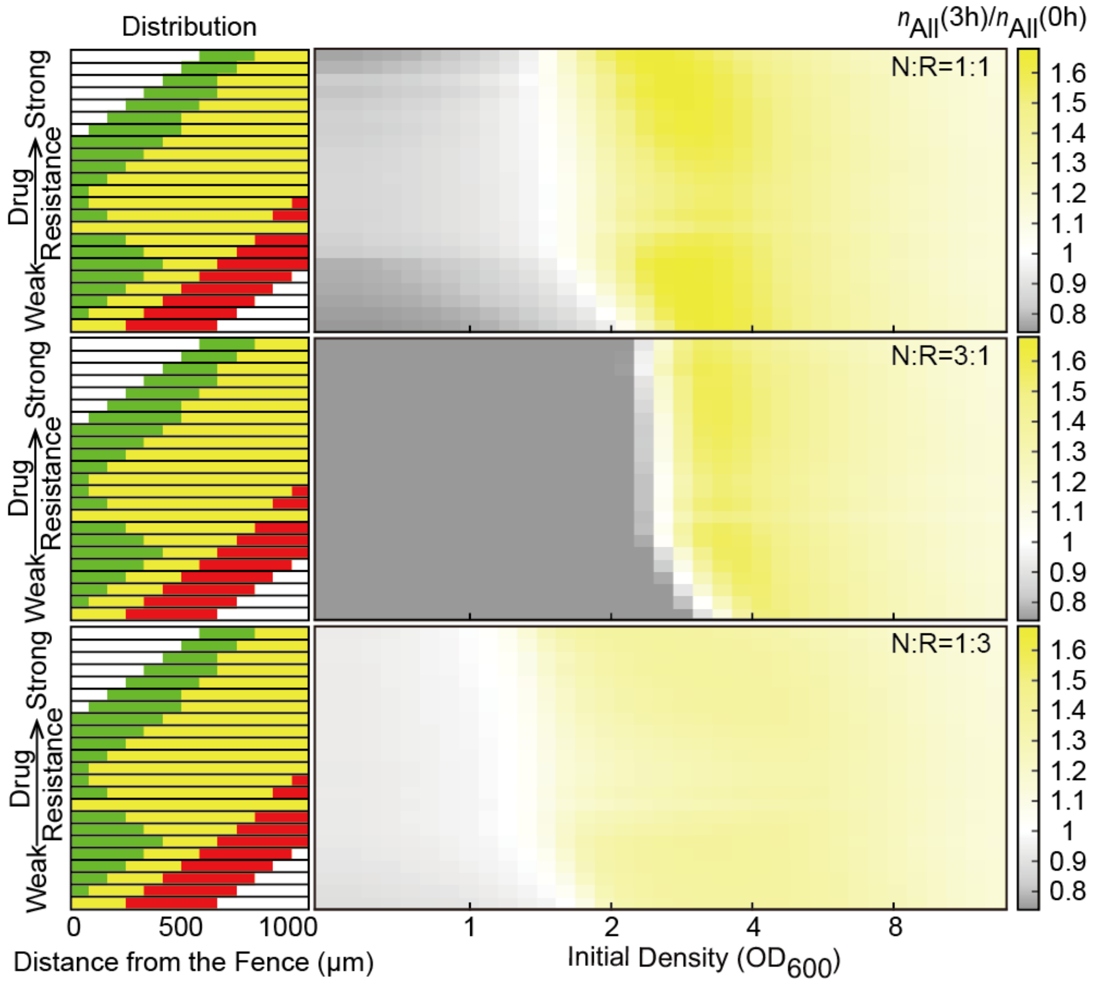

Supplementary Figure 10 Influence of the distribution or N/R ratio on the bacterial community survival rate.

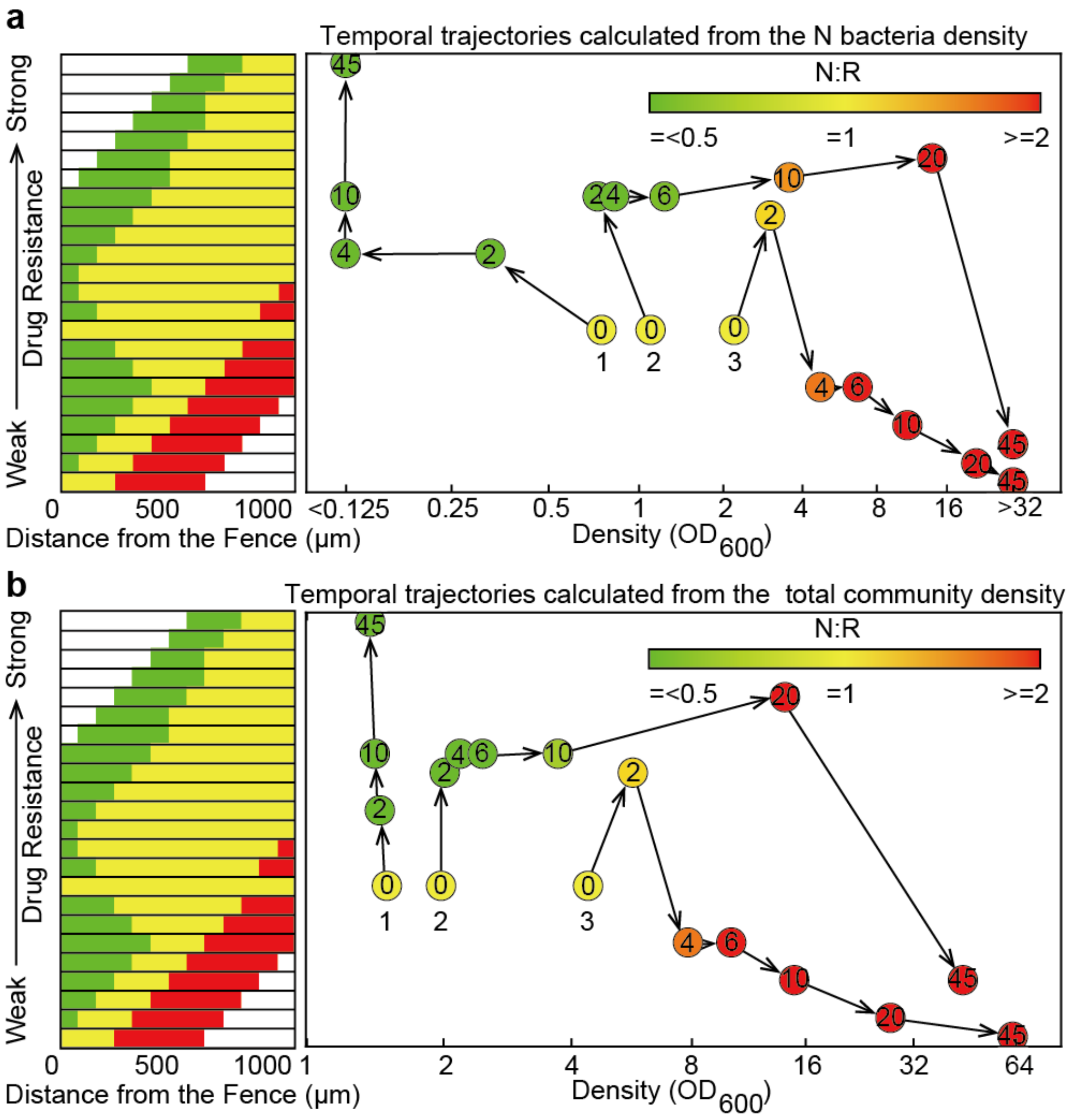

Supplementary Figure 11 Temporal trajectories of the simulations from different initial density samples. The dots and arrows illustrate the temporal trajectories calculated from **a** the N bacteria density or **b** the total community density. The numbers in the circles indicate the time points (h), and the color of the dots indicates the N/R ratio. The numbers below the circles indicate the number of the samples.

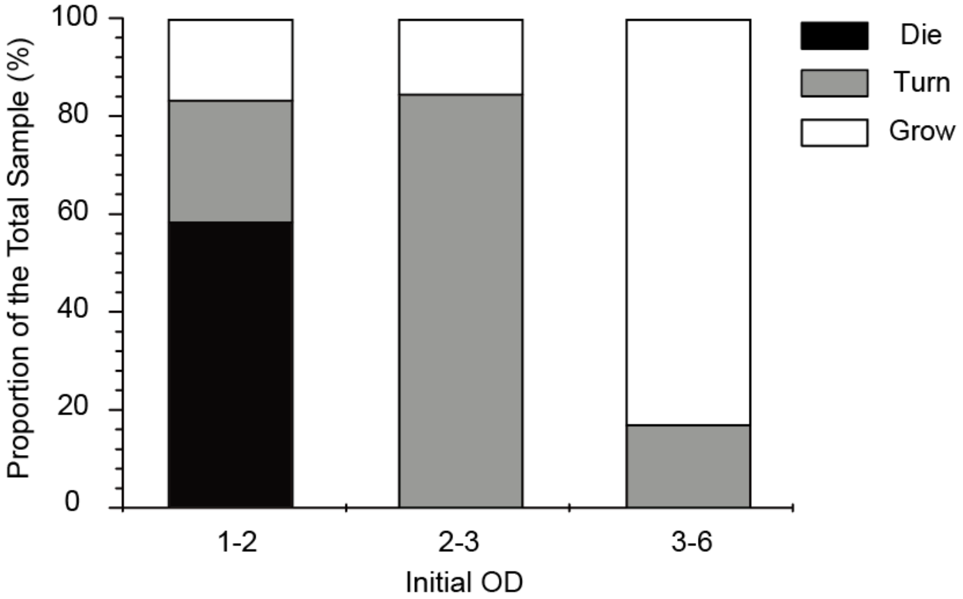

Supplementary Figure 12 Samples that exhibit different track types are presented as percentages of all the samples. The abscissa shows the density range of the sample, and the ordinate is the percentage. Different colors indicate different track types. Black represents the samples that died, and white represents the samples that showed continuous growth. Gray indicates the samples that first died and then grew and thus showed a growth rate inflection point.

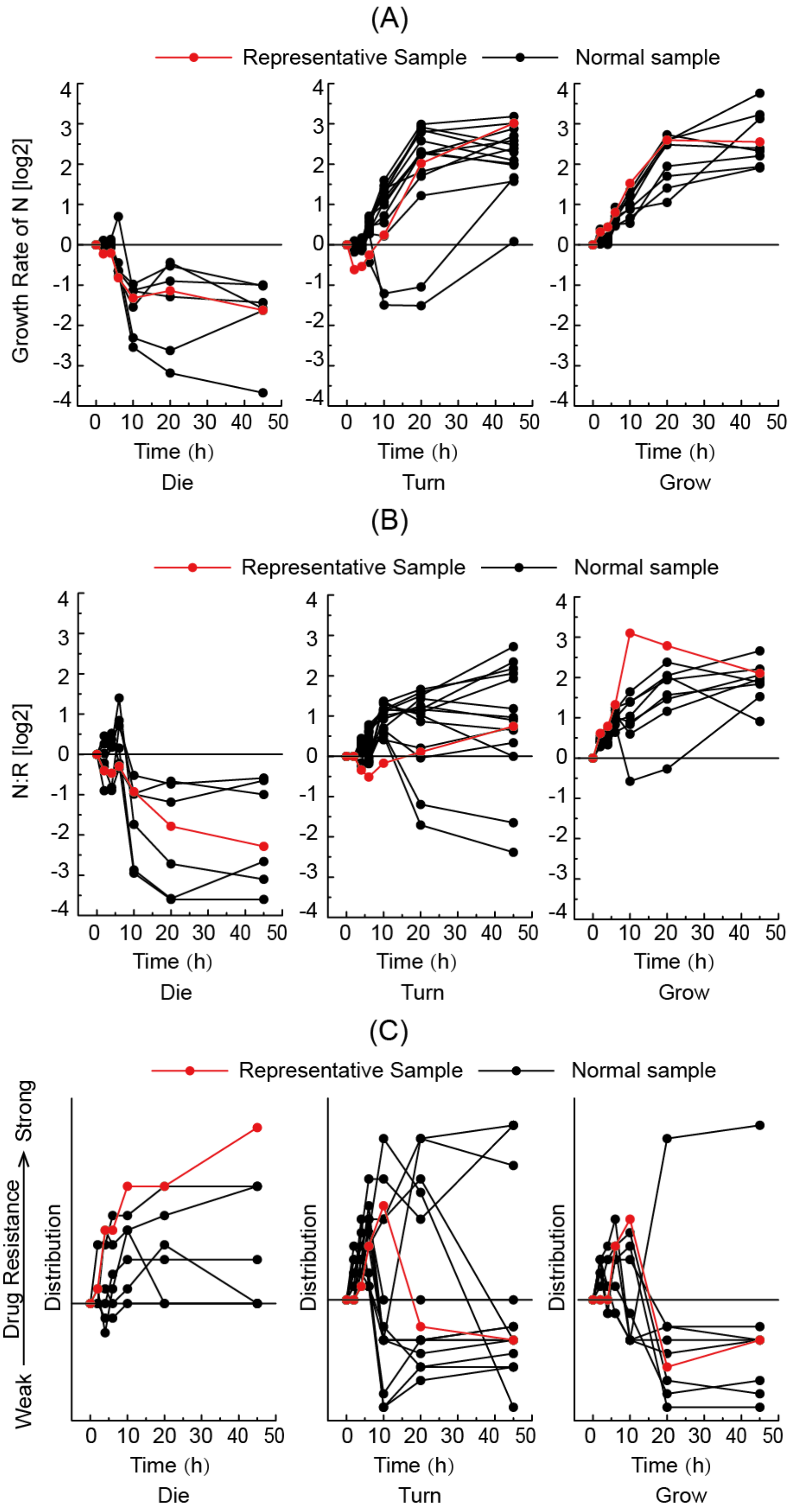

Supplementary Figure 13 Tracks of changes in the community quantity. (A) Growth rate (B) N/R ratio and (C) distribution curves of the nutritional bacteria with different track types. The red dotted lines indicate the selected representative samples, and other samples are indicated by black dotted lines.

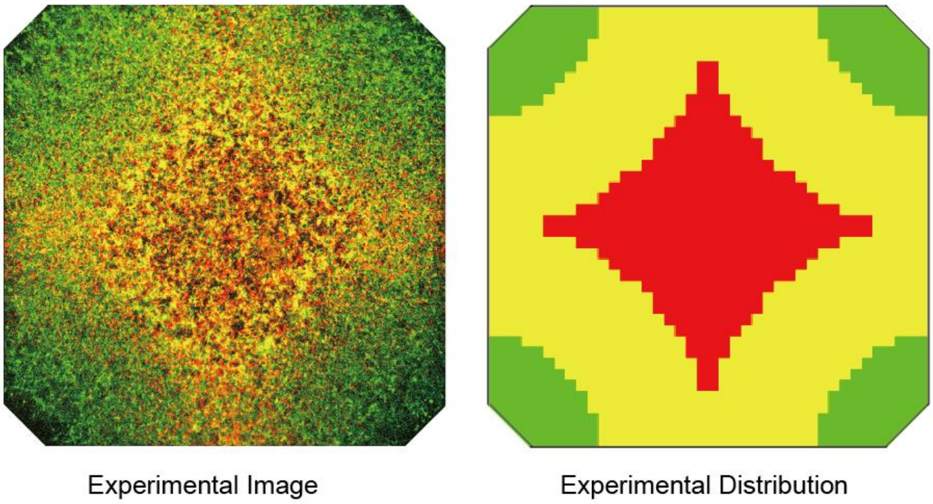

Supplementary Figure 14 Image obtained from the experiment and its corresponding extracted distribution. In the figure, the green color indicates the resistant bacteria, the red color indicates the nutritional bacteria, and the yellow color indicates the overlap between the two bacteria. The depth of the color indicates the density of the bacteria.

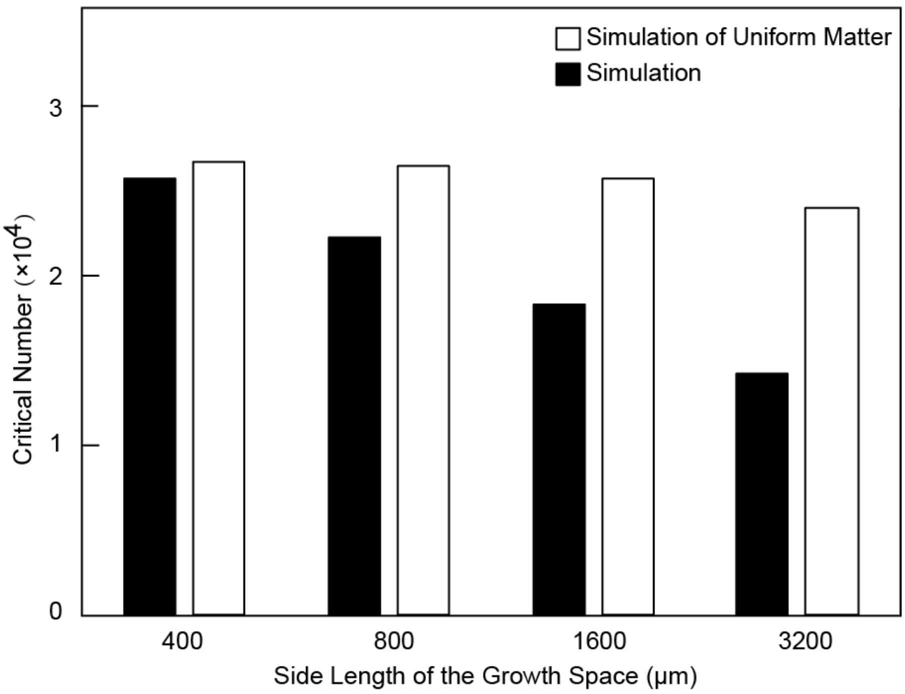

Supplementary Figure 15 Critical numbers (critical density plus system volume) as a function of the spatial size with different simulated conditions.

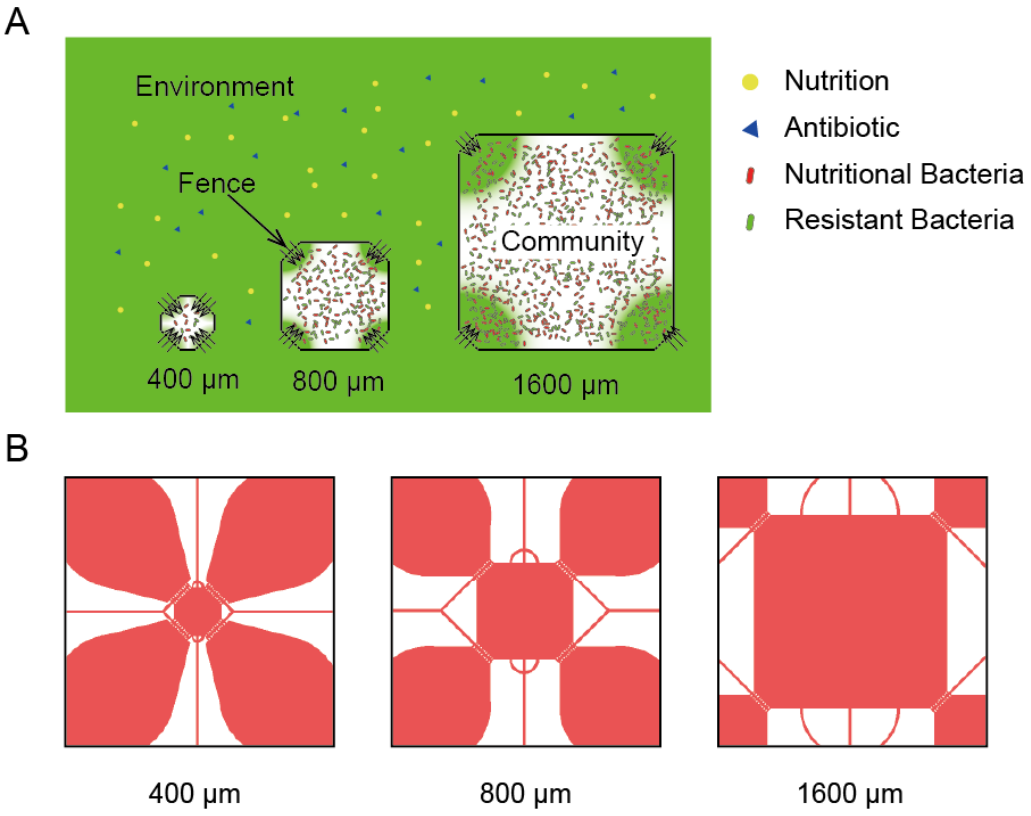

Supplementary Figure 16 Diagrams of the communities grown in differently sized spaces. (A) The three culture areas have different side lengths and the same fences to provide diffusion from the environment. The green area represents the medium, the blue triangles represent the antibiotic molecules, and the yellow triangles represent the lactose molecules. The green and red ellipses indicate the resistant and nutritional bacteria, respectively. (B) To accomplish the above design, chips with culture areas of different sizes were used.

References

1. Si, G., Yang, W., Bi, S., Luo, C. & Ouyang, Q. A parallel diffusion-based microfluidic device for bacterial chemotaxis analysis. *Lab on a chip* **12,** 1389–1394; 10.1039/c2lc21219f (2012).
2. Wang, Y., Ran, M., Wang, J., Ouyang, Q. & Luo, C. Studies of Antibiotic Resistance of Beta-Lactamase Bacteria under Different Nutrition Limitations at the Single-Cell Level. *PLoS ONE* **10,** e0127115; 10.1371/journal.pone.0127115 (2015).
3. Jiang, X. *et al.* Studies of the drug resistance response of sensitive and drug-resistant strains in a microfluidic system. *Integr. Biol.* **6,** 143; 10.1039/c3ib40164b (2014).
