# Supplementary material for: Spatial coordination in a mutually beneficial bacterial community enhances its antibiotic resistance
Source: Commun Biol. 2019 Aug 8;2:301. doi: 10.1038/s42003-019-0533-0 (PMC6687750; doi:10.1038/s42003-019-0533-0)
Supplement: Supplementary file 3 — Description of Additional Supplementary Files [file 42003_2019_533_MOESM3_ESM.docx]

**Description of Additional Supplementary Files**

**File Name**: Supplementary Data 1

**Description**:  Source data for Figures
